# Supplementary material for: Validation of new tablet-based problem-solving tasks in primary school students
Source: PLoS One. 2024 Aug 29;19(8):e0309718. doi: 10.1371/journal.pone.0309718 (PMC11361650; doi:10.1371/journal.pone.0309718)
Supplement: Attachment — Submitted filename: Response to Reviewers_R2.docx [file pone.0309718.s001.docx]

**Point-by-point response to the reviewers**

| **Reviewer-comment** | **Comment** | **Response** |
| --- | --- | --- |
| R 3-1 | In Material and Methods, the description of the Language and Reasoning tasks (page 10 of the revised version) should indicate that they were applied in digital format. This is not deduced from the descriptions offered, and it matters for reproducibility and because the authors indeed allude to a difference in format to explain the lack of predictive relation between Reasoning and the analog GTT tasks. | We added this information to both task descriptions (p. 8 in the current manuscript):  Language: “Participants saw consecutive displays with four pictures **on a tablet screen** and had to point to the picture named by the experimenter.”  Reasoning: “Participants saw a 2x2 matrix **on a tablet screen** containing three pictures and one question mark.”  Thank you! |
| R 3-2 | In the description of the Carrousel task (page 10), the rationale and steps of the second phase are not immediately clear. It is not so common in diagnostic tasks to include probing questions during the task itself (after having had a familiarization or a practice phase). I suggest including some indication about this (especially if the author's goal is that the community can use their tasks upon the information given in this manuscript). Also, if the authors deem it useful to code subject's responses on this phase to complement the assessment, this should be pointed out. | We agree that our description of the second phase of the carousel task was not entirely clear. We therefore rephrased the corresponding paragraph (p. 9):  “In the second experimental phase, the experimenter **removed** the pegboard **(analog modality)** or the tablet **(digital modality)** **temporarily** **and showed** a printed picture of the initial state of the board **to the participants** (i.e., **the state shown in** Fig 1C). **Subsequently**, participants were asked to repeat the task requirements.”  Regarding the second task phase, we coded how well the subject’s responses matched the actual task requirement. Since this study focused on outcome performance, we did not analyze this measure here. However, to make this point more transparent, we integrated the following statement in the task description (p. 9):  “**The accuracy of the participants’ responses was coded** **video-based, but not analyzed in this study.**” |
| R 3-3 | In the Discussion, the authors suggest that the absence of a link between Reasoning and analog GTT performance could have been due to a difference in the tasks' formats (page 17 of the revised version). While I agree that this is plausible, it may lead to think then that the observed relation between Reasoning and digital GTT might have resulted from their shared format (and not because of an underlying cognition). As the reasoning process in the GGT tasks was not measured here (only the quality outcome) a suggestion for a future exploration of these intermediate steps and cognitive tasks may help. | This is a very interesting aspect. We added this aspect to the Discussion (p. 16):  “**This process data provides opportunities to closer investigate the cognitive processes (e.g., reasoning processes) involved in the different problem-solving phases.** Therefore, **the new tasks** provide a significant improvement in the measurement of technical problem-solving strategies beyond qualitative approaches [15, 19] and extend current quantitative accuracy-based research on children's problem-solving [23, 24].”  Thank you very much for this interesting insight! |
| R 3-4 | In the Participants section, as socioeconomic information was not registered, in addition to the statement of no exclusion criteria (page 6), I suggest adding that the sampling area had a heterogeneous population (to compensate for this limitation). | We added the following statement in the Participants section to provide more information on the sample (p. 6):  “**Participants were recruited in a town in southwestern Germany with a heterogeneous and diverse population [31].**” |
| R 3-5 | Regarding software availability, it would be important to know if the authors plan to provide access to their developed tool to the community. | We made this aspect explicit in the Data availability statement (p. 17):  “**The software used in this study will be made available on request by e-mail to the corresponding author.**” |
| R 3-6 | In the Results, the format to present correlation coefficients in the text, separating them by "/" should be checked (pages 13 and 14 of the revised version). | We revisited this formatting style to avoid backslashes and to make clearer which coefficient refers to which task modality.  Thank you very much for your effort! |
